# A Systematic Review of Opioid Use Disorder and Related Biomarkers

**DOI:** 10.3389/fpsyt.2021.708283

**Published:** 2021-08-11

**Authors:** Bianca M. Bryant, Ellen Eaton, Li Li

**Affiliations:** ^1^Department of Psychiatry and Behavioral Neurobiology, University of Alabama at Birmingham, Birmingham, AL, United States; ^2^Division of Infectious Diseases, Department of Medicine, University of Alabama at Birmingham, Birmingham, AL, United States

**Keywords:** opioid use disorder, opioid-related disorders, biomarkers, inflammation, medications

## Abstract

The objective of this systematic review is to examine the relationship between opioid use disorder (OUD) and its related biomarkers, as well as the effects of pharmacotherapy for OUD on biomarkers. The eligibility criteria are the inclusion of human population studies focusing on biomarkers, including the immune system, related to OUD or opioid-related disorders. English, peer reviewed, original research, case studies or case series, and clinical trials were included in this review. Papers were excluded if they met one or more of the following criteria: animal studies, review articles, studies focusing only on OUD or opioid-related disorders without mention of potential biomarkers, studies focusing only on biomarkers and/or the immune system without relating to OUD or opioid-related disorders, and studies that focused on other substance use disorders other than OUD specifically. A PubMed, PsycINFO, and Cochrane databases search on August 25, 2020, yielded 101 results; only 14 articles met inclusion criteria that were included in this review. However, heterogeneity of study definitions and measurements should be noted. Various potential biomarkers indicated systemic, peripheral, and chronic inflammation in patients with OUD or opioid-related disorders. Medications, including buprenorphine and methadone, significantly decreased chronic inflammation in this population. Our results suggest that patients with OUD or opioid-related disorders have potential biomarkers that can be targeted to provide optimal treatment options for this population. A better understanding of potential biomarkers may assist to identify at-risk populations, monitor disease progression and treatment response, and develop therapeutic strategies for OUD.

**Systematic Review Registration:** This review has been registered in PROSPERO (CRD42020202014).

## Introduction

The number of Americans diagnosed with opioid use disorder (OUD) has gradually increased. In 2017, more than 11 million Americans (age 12+) reported misuse of prescription pain relievers/opioids, and an estimated 2.1 million of those met the Diagnostic and Statistical Manual of Mental Disorders-5 (DSM-5) diagnosis of OUD^1^ The current standard of care for OUD treatment is Medications for OUD (MOUD). The Food and Drug Administration (FDA) has approved methadone (full agonist), buprenorphine (partial agonist), and naltrexone (competitive antagonist) for the treatment of OUD^2^ MOUDs can be prescribed or administered alone or in conjunction with behavioral and psychosocial interventions. Despite evidence that patients receiving MOUD are more likely to remain engaged in treatment and less likely to use illicit opioids, widespread adoption of this approach has lagged nationally (1). Approximately 90% of patients with OUD do not receive the recommended addiction treatment (2).

Opioids have been shown to impact both systemic and chronic inflammation in the central nervous system (3, 4). Opioid receptors, mu, delta and kappa, are triggered in response to the presence of opioids and have several other functions, including assisting the immune system (5). These receptors are present on several immune cells (6–10). For example, both T and B lymphocytes express all three opioid receptors. Morphine can alter the function of T and B lymphocytes (11), as well as increase humoral immunity which is antibody mediated immunity (6). In response to opioid use, the body increases the production of cytokines that can affect neurotrophic activity (12). In addition, studies show that individuals with OUD have disproportionately higher rates of adverse childhood experiences, i.e., early life stress (ELS) when compared to the general population, and those adverse childhood experiences are believed to be related to chronic inflammation (13–15). Taken together, these studies suggest that the inflammatory process associated with opioid misuse and OUD may be an important biomarker for the prevention, diagnosis, and treatment of OUD.

Thus, far, studies have not clearly examined the relationship between OUD and biomarkers, including inflammatory biomarkers, nor the effects of MOUD on biomarkers. Determination of these relationships could be a critical component for healthcare providers to provide optimal treatment for people with OUD and prevention to those at greatest risk, i.e., persons living with human immunodeficiency virus (HIV). Therefore, we conducted a systematic review to assess and critically examine various potential biomarkers in OUD and the impact of MOUD on biomarkers to provide vital information and inspire future research.

## Methods

### Search Strategy and Selection Criteria for Systematic Review

Search strategies to identify relevant studies were conducted on August 25, 2020, within three databases: Pubmed, Cochrane, and PsycINFO for articles published with no restrictions to the date range. Search strings for each database contained the same structure and vocabulary (“opioid use disorder” OR “opioid related disorders”) AND (“biomarker” OR “inflammation”). The term “opioid related disorders” in this review is limited to papers that discussed the misuse, use, and abuse of opioids. Endnote X9 was used as the citation manager for all steps of the review, including importation of search results, merging, deduplication, screening and sorting, and full text storage. This review has been registered in PROSPERO (CRD42020202014) to help minimize the risk of bias.

### Papers Meeting the Following Inclusion Criteria Were Included in the Final Selection (*n* = 14)

1. Published on or before August 25, 2020.
2. Human population studies focusing on biomarkers related to OUD or opioid related disorders.
3. English, peer-reviewed, original research, case studies or case series, and medication clinical trials.

**Figure 1:**
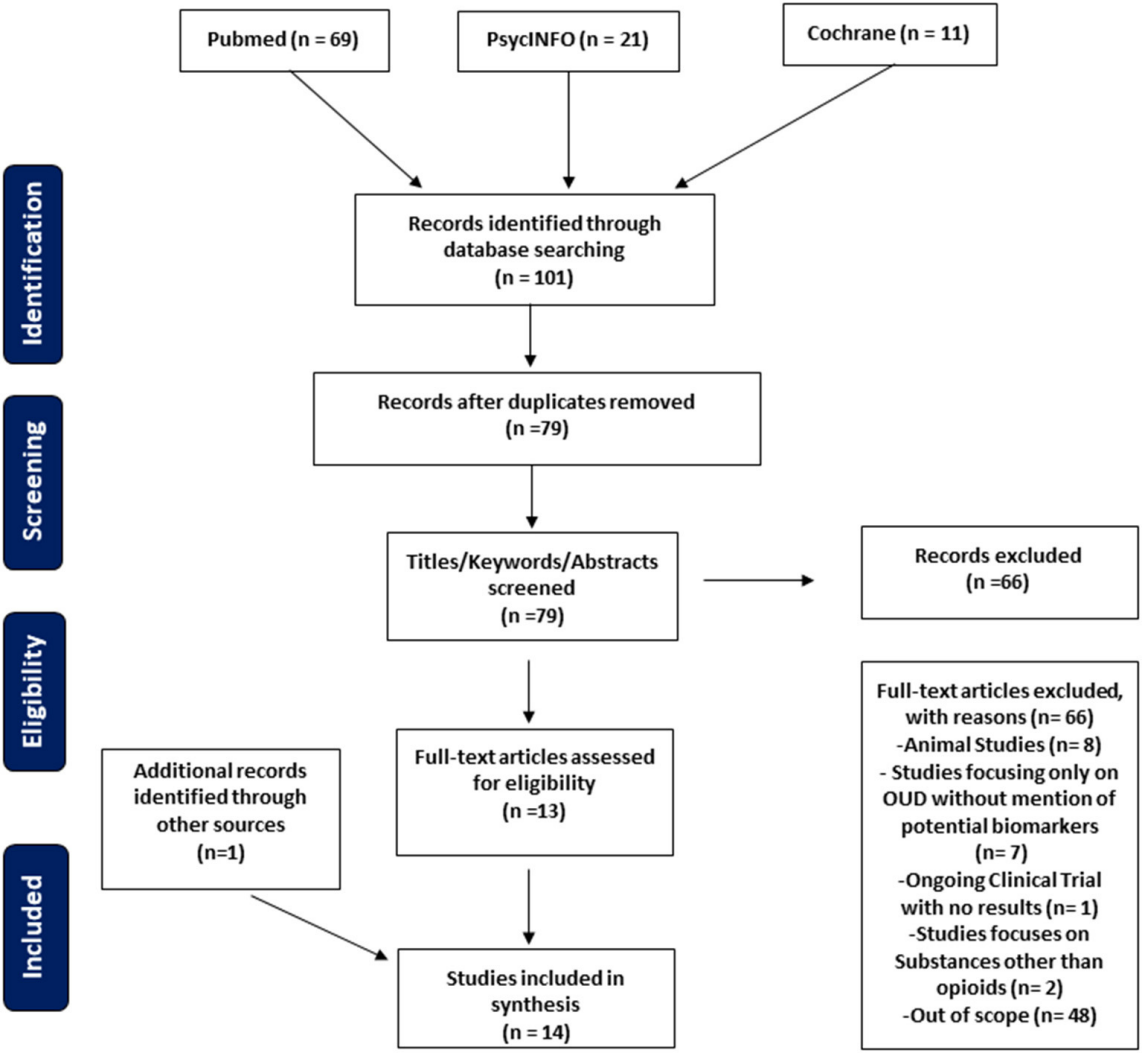
PRISMA flow diagram of literature search and selection of publications.

The search identified 101 citations, and after duplications were removed 79 unique citations were recognized (Figure 1). Exclusion criteria included animal studies, review articles, studies focusing only on OUD or opioid related disorders without mention of biomarkers or the effects on the immune system, studies focusing only on biomarkers and the immune system without relating to OUD, and studies that focused on other substance use disorders other than OUD specifically. Our study focuses on potential biomarkers and the immune system related to OUD. Papers not fitting these criteria were excluded from this review (*n* = 66).

Screening of eligible studies was conducted individually by two authors (BB, LL). Initial screening by title, abstract, and keywords was done independently by both reviewers. During the next stage, full texts were reviewed and accessed independently by the two reviewers. Both authors independently extracted and interpreted data on the included papers. Reviewers then discussed and compared papers deemed eligible by the above criteria and a final agreement was reached on the included papers for this study.

### Data Analysis

Data included study type, time of data collection, study size, biomarker, and relevant findings (Table 1). Excel sheets were used to chart extracted data from each study. The Preferred Reporting Items for Systematic Reviews and Meta-Analyses (PRISMA) guidelines were used as a guide and checklist for the creation of the manuscript (Figure 1).

**Table 1:** Overview of selected studies.

**Lead author**	**Year**	**Type of study based on analysis done**	**Sample size**	**Main findings**
Wang	2017	Cross Sectional Study	• Antisocial personality disorder (*n* = 74) • Substance use disorders (*n* = 168) • Antisocial personality disorder comorbid with substance use disorders (*n* = 438) • Healthy controls (*n* = 81)	- Individuals in the OUD group had significantly higher levels of IL-10 than all other comparison groups.
Shahkarami	2019	Cross Sectional Study	• OUD group • MMT group • Long-term abstinent group • Healthy controls ramya (*n* = 20 in each group)	- The relative expression levels of the preprodynorphin mRNA and prodynorphin peptide in peripheral blood lymphocytes were significantly up-regulated in OUD, MMT, and long-term abstinent groups compared to control subjects. - There was a down-regulation of kappa opioid receptor mRNA in the peripheral blood lymphocytes in all three study groups compared to the controls.
Orum	2018	Retrospective Cohort Study	• OUD group: 61 • Controls: 61	- WBC, lymphocyte count, and red blood cell distribution width were significantly higher in the OUD group. - Percentage of monocyte was significantly lower in the OUD group. - Monocyte to lymphocyte ratio and platelet to lymphocyte ratio were significantly lower in the OUD group.
Guzel	2018	Cross Sectional Study	• OUD group: 51 • Controls: 50	- Hematological effects of heroin/opioid use were seen. - Inflammatory indexes, i.e., neutrophil-lymphocyte ratio and platelet-lymphocyte ratio, were significantly higher in the OUD group compared to the controls.
Moeini	2019	Cross Sectional Study	• Total: 89 • Heroin/OUD with ELS: *n* = 31 (group 1) • Heroin/OUD without ELS: *n* = 32 (group 2) • ELS only: *n* = 26 (group 3)	- Higher levels of soluble tumor necrosis factor receptors were found in groups 2 and 3 compared to group 1. - Increased cortisol and dehydroepiandrosterone sulfate levels in both groups 1 and 2 when compared to group 3. - Also, cortisol levels were significantly higher in group 2 compared to group 1. - Higher WBC counts in both groups 1 and 2 when compared to group 3.
Byrd	2012	Longitudinal and Observational Study(brain bank)	• Total: 46 • Group 1 HIV negative; no opiate use syndrome (*n* = 7) • Group 2 HIV negative; opiate addict (*n* = 7) • Group 3 HIV positive; no opiate use syndrome (*n* = 10) • Group 4 HIV positive; opiate addict (*n* = 13) • Group 5 HIV encephalitis; no opiate use syndrome (*n* = 4) • Group 6 HIV encephalitis; opiate addict (*n* = 5)	- In opiate addicts, there was generally higher baseline expression of CD68 in HIV negatives, and lower expression in HIV and HIV encephalitis, compared to individuals without opiate abuse. - In contrast, for CD163, opiates did not significantly alter its responses to HIV. - HIV effects were variably absent in individuals without opiate abuse.
McGinnis	2019	Electronic Medical Record Based Cohort Study	• Patients with OUD and HIV: 1,964 • Patients with OUD but without HIV: 3,005	- Those with suppressed HIV-1RNA at baseline + consistent negative opioid toxicology had improved CD4. - Those with consistently positive opioid toxicology had declining/worsening CD4. - Those with detectable HIV-1 RNA at baseline and consistent negative opioid toxicology was associated with improvement in CD4, HIV-1RNA, and Veterans Aging Cohort Study Index 2.0.
Jaureguiberry-Bravo	2018	Cross Sectional Study	• N/A	- Buprenorphine treatment decreased neuroinflammation. - Treatment with buprenorphine significantly reduced the C–C motif chemokine ligand 2 induced binding of mature monocytes to brain microvascular endothelial cells which is a major component of the Blood Brain Barrier. - Buprenorphine decreased the ability of monocytes to migrate in response to C–C motif chemokine ligand 2.
Lu	2019	Randomized, Double Blind, Controlled 12-Week Trial	• Patients with OUD at baseline: 104 • Patients with OUD at 12 weeks: 78	- Significant positive correlation between plasma IL-6 levels and MMT outcomes, including the severity of heroin addiction and OUD patient adherence to MMT. - Higher IL6 levels during MMT were associated with poor compliance and dropping out MMT early.
Salarian	2017	Longitudinal Observational	• Patients with OUD • Smoking healthy controls • Non-smoking healthy controls ramya (*n* = 20 in each group)	- Oxidative imbalance in patients with OUD before methadone therapy was shown by a decrease in superoxide dismutase and catalase activity when compared to the non-smoker group.-Opioid misuse increased the oxidative imbalance.-An improvement in oxidative imbalance 14 days after methadone therapy can be seen by the higher levels of superoxide dismutase and catalase activity compared to before methadone therapy.-Lower metallopeptidase 9 and tumor necrosis factor - alpha levels on days 7 and 14 after methadone therapy.
Reece	2016	Cross Sectional Study	• Controls: 51 • OUD group: 233	An increase in adrenocorticotropic hormone/cortisol ratio was observed in female patients with OUD.
Liu	2017	Cross Sectional Study	• Patients with OUD: 20 • Controls: 20	Increased glutamate concentration in the nucleus accumbens.
Kim	2020	Retrospective Cohort Study	• Total: 11,751	Individuals with a vitamin D deficiency were observed to misuse opioids postoperatively and require a higher total opioid dose.
Kemény	2021	Retrospective Cohort Study	• Total: 28,947	Vitamin D deficient or insufficient individuals are more likely to use opioids.

*HIV, human immunodeficiency virus; OUD, opioid use disorder; ELS, early life stress; WBC, white blood cell; MMT, methadone maintenance therapy*.

## Results

### Study Characteristics

This review identified several potential biomarkers that can be used to evaluate OUD. Of the 14 studies included in this review, a total of 10 papers discussed immune system markers. Four papers discussed biomarkers in the peripheral blood that can be used to evaluate OUD. Three papers discussed the changes in immune responses when treated with medications such as methadone and buprenorphine. Biomarkers in patients with OUD were discussed in 3 papers on a cellular level. Additionally, four papers discussed biomarkers related to ELS, vitamin D deficiency, or glutamate concentrations. Among these 14 papers, there were two longitudinal studies, four retrospective studies, seven cross-sectional studies, and one clinical trial. We summarized all of the papers included in this review, including the lead author, the year the paper was published, sample size, and any relevant findings in Table 1.

### Biomarkers in the Peripheral Blood

Changes in cytokines were observed in all patients with OUD relative to a population without the diagnosis of OUD. For example, one study showed a significantly higher expression of interleukin (IL)-10 possibly indicating the body's attempt to circumvent the detrimental effects of the opiates (16). A study found up-regulation of preprodynorphin mRNA and prodynorphin peptide in peripheral blood lymphocytes in patients with OUD or long-term abstinence from OUD or on methadone maintenance therapy, and the down-regulation of the kappa opioid receptor mRNA was also seen in these 3 groups when compared to the healthy controls (17).

Orum and co-authors found hematological changes as a result of opioid misuse. There were significantly higher white blood cells (WBC), lymphocyte count, and red blood cell distribution width in those with opioid misuse relative to those with no history of opioid misuse. In contrast, the percentage of monocytes was significantly lower as well as monocyte to lymphocyte ratio, and platelet to lymphocyte ratio was also significantly reduced in patients with OUD (18). Similarly, hematological effects of heroin/opioid use were also seen in another study (19). In addition, the Guzel group also found that inflammatory indexes, i.e., neutrophil-lymphocyte ratio and platelet-lymphocyte ratio, were significantly higher in patients with OUD compared to healthy controls. A significant difference in median mean platelet volume was found between the OUD group (6.77 femtoliter) and the control group (8.72 femtoliter) (19).

### Biomarkers in OUD and ELS

In a study conducted by Moeini et al., there are three comparison groups: patients using heroin with ELS (group 1), patients using heroin without ELS (group 2), and people not using heroin but with ELS (group 3). Higher WBC counts were observed in both groups 1 and 2 in comparison to group 3 (20). While exploring the relationship between ELS, opioid misuse, and inflammation, higher levels of soluble tumor necrosis factor receptors were found in both groups 2 and 3 compared to group 1 (20). Groups 1 and 2 had significantly higher cortisol and dehydroepiandrosterone sulfate levels than group 3. Also, cortisol levels were significantly higher in group 2 compared to group 1 (20).

### Biomarkers in Patients With OUD and HIV

Patients with OUD have a much higher rate of being infected with HIV, and persons living with HIV and OUD have unique biomarkers. Decreased CD68 in the frontal white matter and thalamus was seen in patients with co-occurring OUD and HIV (21). In contrast, patients with OUD but without HIV had an increased presence of CD68 (21). Opioid misuse was also found to affect the number of CD4 in patients with HIV. McGinnis et al. reported that individuals with suppressed HIV-1 RNA at baseline showed increased CD4 when they had a consistent negative opioid toxicology with an approximate mean of 30 cells per cubic millimeter as opposed to individuals who consistently had positive opioid toxicology with an approximate mean of 61 cells per cubic millimeter (22). Furthermore, patients with detectable HIV-1 RNA at baseline with a consistent negative opioid toxicology were associated with improvement in CD4, HIV-1 RNA, and Veterans Aging Cohort Study Index 2.0 relative to those with detectable RNA but with opioids on toxicology (22).

### Medications for OUD

Buprenorphine may decrease chronic inflammation (23). Specifically, one study showed that buprenorphine decreased neuroinflammation in patients with OUD (24). This study demonstrated that buprenorphine decreases monocyte chemotaxis by inhibiting early signaling by C–C motif chemokine ligand 2, thus not allowing the adherence of monocytes to surface receptors (24). One study showed that IL-6 levels were related to methadone maintenance therapy (MMT) outcomes: higher IL-6 levels were indicative of poor compliance and increased dropout rates (12). Patients with OUD were observed to have increased oxidative stress when compared with people without OUD (25). MMT helped to improve the oxidative imbalance seen in this OUD population (25). The same study on metallopeptidase 9 and tumor necrosis factor-α in patients with OUD showed that after receiving MMT both metallopeptidase 9 (22.21 nanograms per milliliter) and tumor necrosis factor-α (199.96 picograms per milliliter) were decreased to 17.24 nanograms per milliliter and 85.05 picograms per milliliter, respectively, which demonstrates a decrease in chronic inflammation and oxidative stress by MMT (25).

### Additional Biomarkers

Reece's group found that there was an elevation in the adrenocorticotropic hormone/cortisol ratio in females with OUD. There was a significantly higher level of glutamate concentration in the nucleus accumbens in patients with OUD (8.52 Millimoles per liter) in comparison to the control group (7.43 Millimoles per liter) (26). A study found that patients with a vitamin D deficiency were more likely to develop postoperative OUD and required a higher total opioid dose of an additional 98.7 morphine milligram equivalent dose, indicating that vitamin D may be a predictor for the onset of OUD (27). A recent study investigated retrospectively two separate populations, and found that vitamin D deficient or insufficient individuals are more likely to use opioids (28).

## Discussion

This review found evidence for potential biomarkers, including inflammatory and endocrine biomarkers (i.e., lymphocytes, cytokines, and cortisol), associated with OUD and/or opioid related disorders as previously defined in adult populations. Results were heterogeneous, indicating the need for standardization of study measures and further investigation. This review also found evidence for dysregulated inflammation, such as attenuated cytokines, associated with medications (buprenorphine and methadone) used for the treatment of OUD. No research was found investigating the impact of naltrexone, the third FDA approved medication for OUD, on chronic inflammation. The hematologic findings in this review were heterogeneous and should be evaluated further in the future. Due to the heterogeneous findings, more research is warranted to evaluate the relationship between ELS and biomarkers, including chronic inflammation, in the OUD population. Furthermore, there is a lack of research investigating the moderating factors, including age, sex, race/ethnicity, comorbid psychiatric disorders, and other medical illnesses, on these relationships.

The evidence regarding the relationship between OUD and biomarkers merits further study with the overall goal to prevent disease in persons at risk and facilitate earlier detection and treatment in clinical settings for patients with OUD. The biomarkers observed in this review were generally chronic inflammatory factors and hematological effects. While some evidence showed an elevation in IL-10 levels in patients with OUD, this observation seems to be inconsistent with the role of IL-10 as an anti-inflammatory factor (29, 30). Future studies investigating this relationship should aim to use stricter criteria in defining OUD and its comorbidities. Studies also differed in using covariates, which may be an important consideration in future analyses because of the multifactorial characteristics of OUD in patients with- or without- intravenous use of drugs. Covariates, including potential mediators and moderators, need to be explored before independent associations are established.

The studies in this research field are primarily focused on adults even though OUD occurs in pediatric and geriatric populations as well (31–34). These biomarkers may be harder to evaluate in pediatric patients due to stigma, limited access to care, and recruitment challenges, which likely explain the lack of studies done thus far. Research in the pediatric population may also be limited because medications for OUD are underutilized in adolescents, which ultimately may lead to the continuation and intensifying of the disease into adulthood (35). However, research on youth and younger adult populations is a missed opportunity to identify biomarkers that support earlier intervention and better treatment outcomes, which may alleviate the continuation of the disease further into adulthood. Early diagnosis can facilitate harm reduction and prevent the development of infectious diseases such as HIV or hepatitis seen in the adult population with OUD (36, 37).

Our current research findings serve to inspire more study and serve as a foundation for future research on the relationship between OUD and biomarkers. Due to the findings, a concrete conclusion on the risks and benefits experienced by children is unknown and inconclusive in adults. In addition to limited pediatric data, there is a lack of literature on geriatric populations with OUD in general. This highlights a disparity in OUD research and the need for more studies of biomarkers, including immune system and hematological effects, in patients with OUD across the lifespan.

Limitations to the conclusions within this review include varied definitions of OUD diagnosis or opioid misuse, as well as varied measurement and determinants of the immune system and chronic inflammatory factors. Thus, it is hard to compare the effect sizes of each study. Evidence for mechanisms, mediating and moderating factors, was not a focus in any paper, nor did any analyses check for confounding factors. This may have been due to limited statistical power from a small sample size. Race/ethnicity and psychiatric disorders are known risk factors for OUD, however, no papers investigated either as a risk factor. The clinical outcomes of any finding were not discussed within the manuscripts, thus it is unknown whether findings in biomarkers, including those in the immune system, correlate to a higher morbidity risk.

## Conclusion

Individuals with OUD may be at increased risk for dysregulated immune system and hematological processes. However, more research, especially in pediatric and geriatric populations, is warranted to investigate biomarkers in people with OUD to guide prevention, diagnosis, early intervention, and monitor response treatment. Studies must also strive to incorporate demographics such as age, sex, race/ethnicity, and comorbidities in their analyses. More biomarkers, measurements, mechanisms, and factors that may influence the relationship must also be studied to gain a more comprehensive understanding of these relationships. With the growing prevalence of OUD and opioid overdose deaths, it is imperative to understand the role of biomarkers, i.e., the immune system and inflammatory processes, in this specific population.

## Author Contributions

BB and LL collected and analyzed the data, drafted, edited, and approved the final manuscript. EE reviewed, edited, and approved the final manuscript. All authors contributed to the article and approved the submitted version.

## Conflict of Interest

The authors declare that the research was conducted in the absence of any commercial or financial relationships that could be construed as a potential conflict of interest.

## Publisher's Note

All claims expressed in this article are solely those of the authors and do not necessarily represent those of their affiliated organizations, or those of the publisher, the editors and the reviewers. Any product that may be evaluated in this article, or claim that may be made by its manufacturer, is not guaranteed or endorsed by the publisher.

## Footnotes

^1^ https://addiction.surgeongeneral.gov/sites/default/files/surgeon-generals-report.pdf

^2^ https://journals.lww.com/hrpjournal/Fulltext/2015/03000/Medication_Assisted_Treatment_of_Opioid_Use.2.aspx

## Abbreviations

HIV: human immunodeficiency virus
RNA: ribonucleic acid
OUD: opioid use disorder
IL: interleukin
ELS: early life stress
WBC: white blood cells
mRNA: micro ribonucleic acid
MMT: methadone maintenance therapy.
